# A Canterbury Tale

**Published:** 1890-09-27

**Authors:** 


### Passing Topics.

### A CANTERBURY TALE.

That the authorities of the Canterbury Hospital have decided to discontinue the quarterly meetings of governors and others, because nobody has thought well for a long time past to attend them, demonstrates that even provincial institutions cannot command the unremitting solicitude of those who might be reasonably expected to be most interested in them. It may also be said to prove that the affairs of the charity are moving in channels smooth enough to prevent a superabundance of attention, and so far there is in this lack of concern something in the nature of testimony—negative, it is true, but still testimony—to efficiency.

Representatives of metropolitan institutions not infrequently feel disposed to envy their country brethren that neighbourly interest which they themselves are either denied or must be content to receive in infinitesimal portions. It is only here and there that a metropolitan hospital is recognised as deserving well of its neighbourhood. The parochial mind not seldom is inclined to argue that the existence of a hospital is an evil rather than a blessing, inasmuch as it attracts to itself alien, halt, and impotent folk, who settle round about it and by ultimately becoming chargeable to the parish, serve to increase the imposts it is but natural to resent. This is a view which was publicly put forward not long ago at a meeting to organise opposition to the Bill projected to relieve hospitals from local rates. But in enlightened country towns where the claim of the sick is recognised by the wealthier classes as emanating from the unfortunate of their own kith and kin, and within the sanctified precincts of a provincial cathedral city, better views were supposed to prevail. The cold and mechanical performance of duty, too often the prelude to neglect, was believed to give place to a warm sensibility which preserved philanthropy from perfunctoriness and inspired even committee men with enthusiasm.

We are afraid this belief must now give way to the conviction that human nature is much the same everywhere, and that the constant and never failing co-operation, such as we would fain believe is truthfully pourtrayed in the pages of the annual report, is but a delightful fiction—the survival of simpler times, now vanished like the pixies of Devon. Look at the names upon the title page! What an imposing array it is; and we are told, all these notabilities, great and small, are "earnestly endeavouring," or they are making "strenuous efforts" on behalf of the institution. One would suppose the object in view must be a stupendous one, yet it is often nothing more than to keep alive some organisation with petty ambitions altogether unworthy of the field of usefulness lying before it, sometimes only making visible the darkness of its neglect, and whose total expenditure would not appreciably tax the pockets of the multitude of persons asserting to be labouring in its interests. Earnestness and sincerity—these are the great wants of the time, in charitable, as in other, matters!

The penetrating vision of the author of the "Scarlet Letter" has not allowed this to escape him. It may be remembered how, in another of his romances, he tells us that among the presumptive engagements of the dead Judge Pyncheon, was the intention," should all the other business of the day be reasonably got through with, to attend the meeting of a charitable society, the very name of which, however, in the multiplicity of his benevolence, is quite forgotten." Keen as the satire is, and the more keen when we recall the character of the man of whom it was uttered, can we complain of its severity? The Canterbury Hospital has furnished the latest audible evidence to its truth, but the silent witnesses—are they not legion?

Agricola.

